# Pediatric sensorimotor cortical responsiveness to intracerebral stimulation during stereoelectroencephalographic monitoring: Age effects and area specificity

**DOI:** 10.1002/epi.70231

**Published:** 2026-04-03

**Authors:** Giulia Nobile, Laura Tassi, Marta Ponzano, Piergiorgio d'Orio, Alessandro Consales, Luca Bosisio, Stefano Francione, Gabriele Arnulfo, Maria Pia Sormani, Lino Nobili, Roberto Mai

**Affiliations:** ^1^ Child Neuropsychiatry Unit full member of European Reference Network EpiCARE, IRCCS (Istituto di Ricovero e Cura a Carattere Scientifico) Istituto Giannina Gaslini Genoa Italy; ^2^ “Claudio Munari” Epilepsy Surgery Center–ASST Great Metropolitan Hospital Niguarda Milan Italy; ^3^ Department of Health Sciences University of Genoa Genoa Italy; ^4^ Pediatric Neurosurgery Unit, IRCCS Istituto Giannina Gaslini Genoa Italy; ^5^ Department of Neurosciences, Rehabilitation, Ophthalmology, Genetics, and Maternal and Child Health University of Genoa Genoa Italy; ^6^ Department of Informatics, Bioengineering, Robotics, and System Engineering University of Genoa Genoa Italy; ^7^ IRCCS Ospedale Policlinico San Martino Genoa Italy

**Keywords:** cortical responsiveness, developmental neurophysiology, network maturation, operculum, premotor cortex, SEEG

## Abstract

**Objective:**

This study was undertaken to determine how age influences clinical responsiveness to intracerebral electrical stimulation (IES) in children across primary and secondary sensorimotor cortices and to assess age effects on response complexity and area‐specific responsiveness.

**Methods:**

A retrospective cohort of pediatric (≤16 years) stereoelectroencephalographic procedures was analyzed. A total of 1750 IESs were delivered in 33 implantations (31 patients). Precentral and postcentral gyri were considered as primary areas; secondary areas included premotor cortex (PREMOT), supplementary sensorimotor area (SSMA, including presensorimotor area), anterior/central cingulate (ACC), and pre‐/postcentral opercula (PREC‐OP and POSTC‐OP). Clinical responses were classified as simple motor, sensory, language, or undefined. Complexity was coded as integrated (one coordinated action) or multiple (signs from different domains occurring together). Mixed‐effects models were adjusted for antiseizure medication load, cognitive level/neurodevelopmental delay, and stimulation frequency (1 vs. 50 Hz). Age was modeled continuously (per year) and categorically (≤9 vs. >9 years).

**Results:**

Clinical response rate increased with age in the overall cohort (per‐year incidence rate ratio [IRR] = 1.11, 95% confidence interval [CI] = 1.03–1.21, *p* = .007). Categorical modeling showed a higher rate in subjects aged >9 years (IRR = 1.89, 95% CI = 1.02–3.51, *p* = .044). Integrated responses did not vary with age, whereas multiple responses were more likely in older children (overall adjusted odds ratio [OR] = 3.65, 95% CI = 1.24–10.77, *p* = .019; without postdischarge: adjusted OR = 5.30, 95% CI = 1.38–20.36, *p* = .015). Using PREMOT as reference, all other regions were more responsive, forming two clusters: lower (ACC, PREC‐OP/POSTC‐OP) and higher (SSMA, precentral, postcentral); SSMA showed rates comparable to primary cortices.

**Significance:**

Pediatric cortical responsiveness to IES rises with age and is independent of medication burden, cognitive level, and stimulation frequency. Older children also show more multidomain responses, consistent with developing large‐scale integration. These results can inform age‐aware stimulation mapping in clinical practice.

**Key points**

- Clinical responsiveness to IES increases with age in children.
- “Multiple” (cross‐domain) responses are more frequent at >9 years.
- Integrated motor actions do not show age dependence.
- Findings are independent of ASMs, cognition, and frequency.

## 1 INTRODUCTION

The first intracerebral electrical stimulations (IESs) were conducted by Bartholow in 1874 during the surgical removal of a scalp epithelioma.^1^ Since then, IES has become a technique of growing interest, particularly in the field of epilepsy surgery.^2^ IESs are particularly useful in defining the epileptogenic zone (EZ) and in delineating the boundaries and relationships of eloquent cortical areas (e.g., language, visual, or motor regions), especially when close to regions of surgical interest. Recently, a certain level of consensus has emerged regarding the finding that intracerebral (IC) electrodes, typically used for intracranial electroencephalography (EEG) with stereotactic electrodes (stereo‐EEG [SEEG]), are more effective for presurgical evaluation of epilepsy than subdural electrodes.^3^ Despite the increasing use of IC electrodes, no internationally recognized guidelines currently exist to specify the optimal stimulation parameters,^4^, ^5^, ^6^, ^7^ although recent American recommendations provide technical standards for clinical practice.^8^ Although the heterogeneity of these parameters—such as pulse type (mono‐ or bipolar), frequency, intensity, and duration—is partly determined by the type of SEEG implant and the patient's clinical condition, it is also influenced by the experience of the single epilepsy center. This leads to data that are not easily comparable across centers, posing an additional challenge to the development of international guidelines. Specifically, in the pediatric field, limited studies have examined the response to IES. These few studies, along with intriguing findings on age‐related variations in ictal semiology, suggest that IES yield effects that vary with the tissue's degree of maturation.^8^, ^9^ Based on these premises, we aimed to enhance our understanding of IES in the pediatric population, evaluating the primary and secondary sensory–motor areas.^10^, ^11^ We initially focused on the primary sensory–motor areas, including the precentral and postcentral gyri, together with their mesial extensions in the paracentral lobule. Secondary sensory–motor areas were defined according to cytoarchitectonic features. These include the premotor cortex, the supplementary motor area, and the anterior and mid/central cingulate cortices.^10^, ^12^, ^13^ In line with cytoarchitectonic and functional accounts that place the frontal (precentral) operculum within ventral premotor cortex and the parietal (postcentral) operculum within secondary somatosensory cortex, we grouped the precentral and postcentral opercula among secondary sensory–motor areas.^10^, ^12^, ^14^, ^15^ We hypothesized that age influences the probability and phenomenology of IES‐elicited clinical responses within primary and secondary sensorimotor cortices. SEEG centers recognize that IES sessions often differ between adolescents and children. There is a strong clinical impression that responsiveness changes across development, with a transitional age window—roughly between 6 and 10 years—during which the propensity to show adultlike responses seems to emerge. Clinically, pediatric SEEG mapping also suggests that younger children often show fewer or less complex observable responses, beyond differences in cooperation and stimulation settings. Therefore, although modeling age as a continuous predictor as our primary approach, we also explored whether the age–response relationship showed a nonlinear transition point that could be clinically meaningful for interpreting stimulation mapping result.

## 2 MATERIALS AND METHODS

### 2.1 Patients

We retrospectively analyzed patients with focal epilepsy who underwent SEEG for refractory focal epilepsy over a 153‐month period between January 2011 and December 2023 at the “Claudio Munari” Epilepsy Surgery Center of Niguarda Hospital, Milan (Italy), and the Epilepsy Center of IRCCS Giannina Gaslini.

We included only patients aged ≤16 years who underwent SEEG implantation involving the primary or secondary sensory–motor areas (see the subsequent section for definitions of areas of interest). All patients were receiving antiseizure medications (ASMs) at the time of stimulation, as monotherapy or polytherapy. IC stimulation sessions were performed under the ongoing clinical ASM regimen; ASM management during SEEG followed routine clinical care and was not driven by the stimulation protocol. We gathered 33 implantation schemas, that is, comprehensive plans outlining the specific electrode trajectories and placement configurations used during SEEG, from 31 patients (including two patients who were reimplanted). All patients had normal neurological examinations and sufficient cognitive and collaborative abilities to facilitate a comprehensive clinical evaluation during IES. All patients and caregivers were fully informed of the aims of the SEEG recording and stimulation procedures and gave their written informed consent in agreement with the Declaration of Helsinki. The current retrospective study received the approval of the Niguarda Hospital ethics committee (ID 939–12.12.2013).

### 2.2 IC electrode implantation and electrical stimulation paradigm

Thirty‐one patients were chronically implanted with semirigid platinum/iridium IC electrodes. Twenty‐nine received electrodes from Dixi Medical and two from Alcis/Temis Santé; in both cases, electrodes had a diameter of .8 mm, a contact length of 2 mm, and an intercontact distance of 1.5 mm. Electrodes included 5–18 recording contacts. Twenty implantations were on the right side, 11 on the left, and two bilaterally, with 4–18 electrodes implanted in each case. The number of electrodes and implantation sites was determined based on noninvasive anatomical, electrical, and clinical data collected during the noninvasive presurgical phase. This approach enabled designing a tailored exploratory strategy that aligned with each patient's hypothesized EZ. Electrode positions were measured by coregistering the postimplant computed tomography (O‐arm 1000 system, Medtronic) to the preimplant magnetic resonance imaging (MRI) by means of the FLIRT software.^16^ The location of every single lead was assessed using FreeSurfer,^17^ 3D Slicer,^18^ and SEEG Assistant.^19^

Electrical bipolar stimulations of two adjacent contacts were carried out at the following:

- Low frequencies: 1 Hz, pulse width = 1–3 ms, 30 s, .4–7 mA.
- High frequencies: 50 Hz, pulse width = 1 ms, 1–5 s, .2–5 mA.

Electrical stimulations were performed by delivering monophasic rectangular electrical stimuli of alternating polarity (IRES 600 CH electrical stimulator, Micromed or OSIRIS NeuroStimulator, Inomed) to map functionally eloquent regions and reproduce ictal manifestations. These parameters were used to avoid any tissue injury (predominantly charge density per square pulse < 55 µC/cm^2^).^5^ Derived stimulation dose metrics (charge per phase and charge density per phase, calculated from the electrode contact geometry) are reported in Table S1.

### 2.3 Data collection: Clinical and electrophysiological responses

For each stimulation, we collected both the clinical responses, defined as the observable semiological effects induced by IES, and the electrophysiological responses. Area distribution was defined as the number of IESs delivered in each predefined cortical area, expressed as a proportion of all IES in that age group. For the purposes of this study, we analyzed the electroclinical responses during IES recorded at the level of the following cortical areas:

1. Primary sensory–motor areas: precentral gyrus (PREC) and postcentral gyrus (POSTC).
2. Secondary sensory–motor areas: premotor cortex (PREMOT), supplementary sensory–motor area including the presupplementary part (SSMA), anterior and central cingulate cortex (ACC), precentral operculum (PREC‐OP), and postcentral operculum (POSTC‐OP).

We excluded from the analysis those IES sessions that met any of the following criteria:

- Conducted on electrodes located in lesions visible on MRI.
- Conducted on electrodes located within a lesion or recording pathological activity (i.e., interictal epileptiform abnormalities).
- Inducing diffuse postdischarge (PD), defined as diffuse electrical activity following the cessation of stimulation (with or without associated clinical signs).
- Reproducing part or the entire habitual ictal symptomatology.
- Conducted at contact pairs situated in white matter tracts.
- Conducted between contacts placed in different gyri.

All other IES sessions were included, regardless of whether they elicited evoked responses or focal PD. Focal PD was defined as PD restricted to the stimulated contacts and/or adjacent contacts within the same gyrus (Figure S1); PD extending to contacts in a different gyrus (e.g., motor‐to‐sensory) or to other electrodes was classified as propagated and excluded. We retained stimulations associated with focal PD because PD is commonly reported as an indicator that stimulation effectively engaged cortical tissue (i.e., reached an activation threshold), as reported by Trebuchon.^20^

Throughout the whole stimulation session, patients were seated in bed while the examiner assessed them by evaluating both objectively quantifiable factors, particularly through targeted motor assessments of the examined cortical regions, and subjective components.

Each IES was analyzed from both the electrophysiological (PDs) and clinical (semiological effects) perspectives. The clinical response rate was defined as the proportion of stimulations that elicited an observable clinical sign temporally related to the IES.

Those that elicited a physiological response were further classified by detailing the clinical manifestations observed during stimulation. Each manifestation was evaluated by identifying and defining its individual clinical signs—both objective and subjective—that together constituted the response. All clinical signs were subsequently categorized into the following classifications:

1. Simple motor clinical signs were defined as elementary motor signs, including muscle jerks, dystonic movements, extension, flexion, contraction, deviation, abduction, rotation, combined oculocephalic rotation, oral fissure stretching, loss of segmental tone, segmental slowing, segmental arrest, and tremor.
2. Sensory clinical signs encompassed any sensations experienced by the patient: auditory illusion, visual illusion, imbalance, pain, nausea, paresthesia, proprioception, pulsation, vibration, auditory hallucination, and gustatory hallucination.
3. Language clinical signs included any phonological modifications such as anarthria, dysarthria, naming difficulties, and altered timbre.
4. Undefined clinical signs referred to nonlocalizing subjective manifestations that are challenging to classify because they are inconsistent or not specific enough to be categorized as a defined clinical response, such as dizziness, lightheadedness, difficulty breathing, or dyspnea without objective signs.

In case of more than one clinical sign, we defined the quality of clinical response as

1. *Integrated response*: referred to a coordinated, purposeful motor output that involved multiple systems or body parts working together in a complex, often context‐appropriate way. Included movements like grasping, open–close hand movements, laughter, automatisms, changes in facial expressions, stopping and slowing down globally, and changing posture.
2. *Multiple response*: the response was defined as an aggregate of different clinical signs (motor simple ± sensory ± language ± undefined) without constituting an integrated motor behavior.

### 2.4 Statistical analysis

Descriptive statistics were reported as mean (SD) or median (interquartile range [IQR]) for continuous variables and as *n* (%) for categorical variables. To study the impact of age and of site on the response, mixed‐effects models were performed to account for the two levels (SEEG and patient) while adjusting for prespecified confounders, including ASM load (number of concomitant ASMs at the time of stimulation: 1, 2, 3, or 4), neurodevelopmental impairment (binary; presence vs. absence of clinically documented neuropsychomotor delay and/or intellectual/developmental disability based on the most recent clinical/neuropsychological assessment in the medical record), and stimulation frequency (1 vs. 50 Hz). Specifically, negative binomial regression was used to model the number of responses, whereas logistic regression was performed for the binary outcomes (multiple response and integrated motor clinical response). We further investigated whether a specific age threshold could distinguish differential response outcomes in our sample. Specifically, we graphically observed the locally weighted regression of number of responses on age and we identified the optional cutoff in discriminating presence of binary outcomes by age according to the Liu criterion.^21^ To determine whether the distribution of IES across the various cortical areas of interest was homogeneous in the two age groups, a contingency table was constructed, and the chi‐squared test was used independently. Furthermore, using Fisher exact test or Pearson chi‐squared, the relationship between age and the hertz and voltage of the IES that caused focal PD was examined. A two‐sided *α* < .05 was considered statistically significant. All statistical analysis was performed using Stata version 18.0 (Stata Corporation).

## 3 RESULTS

### 3.1 Quantitative and qualitative analysis of IES

Tables 1, 2, 3 summarizes the features and localization of IES.

**TABLE 1:** Electrical features of total intracerebral electrical stimulation.

	Entire population	%
Total IES	1750	–
Total SEEG implantation	33	–
IES, median (IQR) per SEEG	51 (28–70)	–
Total IES 1 Hz [total IES%]	1291	73.77%
Total IES 50 Hz [total IES%]	459	26.23%
IES without PD [total IES%]	1641	93.77%
IES 1 Hz without PD [IES without PD%]	1286	78.37%
IES 50 Hz without PD [IES without PD%]	355	21.63%
IES with focal PD [total IES%]	109	6.23%
IES 1 Hz with focal PD [IES with focal PD%]	5	4.59%
IES 50 Hz with focal PD [IES with focal PD%]	104	95.41%
IES 1 Hz without PD, mA, median (IQR)	5 (.4 –7)	–
IES 1 Hz with focal PD, mA, median (IQR)	3 (3 –5)	–
IES 50 Hz without PD, mA, median (IQR)	2 (.4–4)	–
IES 50 Hz with focal PD, mA, median (IQR)	3 (.2 –5)	–

*Note*: The denominator to which the percentage pertains is enclosed in square brackets. Abbreviations: IES, intracerebral electrical stimulation; IQR, interquartile range; PD, postdischarges; SEEG, intracranial electroencephalography with stereotactic depth electrodes.

**TABLE 2:** Age‐related differences in electrical parameters of intracerebral electrical stimulation in the two groups, >9 versus ≤9 years old.

	≤9 years	%	>9 years	%
Total IES	555	–	1195	–
Total SEEG implantation	11	–	22	–
IES, median per SEEG (IQR)	46 (28–70)	–	51.50 (26–76)	–
Total IES 1 Hz [total IES%]	395	71.17%	896	74.98%
Total IES 50 Hz [total IES%]	160	28.83%	299	25.02%
IES without PD [total IES%]	505	90.99%	1136	95.06%
IES 1 Hz without PD [IES without PD%]	392	77.62%	894	78.70%
IES 50 Hz without PD [IES without PD%]	113	22.38%	242	21.30%
IES with focal PD [total IES%]	50	9.01%	59	4.94%
IES 1 Hz with focal PD [IES with focal PD%]	3	6.00%	2	3.39%
IES 50 Hz with focal PD [IES with focal PD%]	47	94.00%	57	96.61%
IES 1 Hz without PD, mA, median (range)	5 (2–5)	–	5 (.4–7)	–
IES 1 Hz with focal PD, mA, median (range)	4 (3–5)	–	5 (3–5)	–
IES 50 Hz without PD, mA, median (range)	1 (.4–3)	–	2 (.4–4)	–
IES 50 Hz with focal PD, mA, median (range)	3 (.2–5)	–	2 (1–4)	–

*Note*: The denominator to which the percentage pertains is enclosed in square bracket. Abbreviations: IES, intracerebral electrical stimulation; IQR, interquartile range; PD, postdischarges; SEEG, intracranial electroencephalography with stereotactic depth electrodes.

**TABLE 3:** Localization of intracerebral electrical stimulation.

	Total	%	≤9 years	%	>9 years	%
PREC	253	14.46%	97	17.48%	156	13.05%
POSTC	170	9.71%	44	7.93%	126	10.54%
PREC‐OP	227	12.97%	64	11.53%	163	13.64%
POSTC‐OP	201	11.49%	68	12.25%	133	11.13%
PREMOT	461	26.34%	159	28.65%	302	25.27%
ACC	311	17.77%	97	17.48%	214	17.91%
SSMA	127	7.26%	26	4.68%	101	8.45%
Total IES	1750	100.00%	555	100.00%	1195	100.00%

Abbreviations: ACC, anterior and central cingulate cortex; IES, intracerebral electrical stimulation; POSTC, postcentral gyrus; POSTC‐OP, postcentral operculum; PREC, precentral gyrus; PREC‐OP, precentral operculum; PREMOT, premotor cortex; SSMA, supplementary sensory–motor area including the presupplementary part.

As anticipated in Materials and Methods, the age effect was initially explored as a continuous predictor using locally weighted regression, which showed a clear departure from linearity with an inflection around 9 years (Figure S2). Based on this data‐driven pattern, and to facilitate interpretation, we subsequently categorized IES into two age groups (>9 vs. ≤9 years). In parallel, age effect was examined using nondichotomized approaches (per‐year/continuous modeling) to confirm the robustness of the findings. This data‐driven threshold was associated with a higher likelihood of observing a clinical response during IES. Moreover, at this cut‐point, the probability of being older than 9 years in the presence of outcomes “integrated response” and “multiple response” was .73 and .86, respectively.

The results of these analyses are presented in Sections 3.2 and 3.3. IES included 555 conducted in the ≤9‐year category (11 SEEG) and 1195 in the >9‐year category (22 SEEG), yielding an average of 50.45 IESs for SEEG in the ≤9‐year group against 54.32 in the >9‐year group. The electrical paradigm used included frequencies of 1 Hz and 50 Hz, with comparable distribution proportions across the ≤9‐year (IES 1 Hz 71.18% vs. 50 Hz 28.82%) and >9‐year groups (IES 1 Hz 74.9% vs. 50 Hz 25.1%). IESs that induced a focal PD were 109, whereas the IESs that did not induce PD were 1641.

The elicited focal PDs were uniformly distributed across the ≤9‐year and >9‐year groups, with the majority recorded during the IES at 50 Hz in both age categories. There were only five IESs that induced a focal PD at 1 Hz (*n* = 3 in ≤9‐year vs. *n* = 2 in >9‐year group, *p* = .659). Concerning the amperages used, during the IES at 1 Hz, there were no notable discrepancies in the amperage employed between the IESs accompanied by focal PD and those not accompanied by PD. We noted a minor variation in current intensity across 50‐Hz stimulations; nevertheless, IESs that elicited focal PDs occurred at higher currents than non‐PD IESs in both age groups, with no significant difference between ≤9‐year and >9‐year groups. The distribution of stimulation sites by cortical area was comparable between age groups, except for SSMA, which was more frequently sampled in >9‐year than ≤9‐year subjects (8.45% vs. 4.68%, *p* = .045). Moreover, each area of interest was stimulated at frequencies of 1 Hz and 50 Hz throughout the two age cohorts.

### 3.2 Response rate related to age

We analyzed 1750 IESs, 1195 in the >9‐year group and 555 in the ≤9‐year group (Table 1). In the overall cohort (IES with and without focal PDs), the clinical response rate increased with age (per‐year incidence rate ratio [IRR] = 1.11; 95% confidence interval [CI] = 1.03–1.21, *p* = .007). When age was modeled categorically (>9 years vs. ≤9 years), the response rate was higher in >9‐year subjects (IRR = 1.89, 95% CI = 1.02–3.51, *p* = .044).

**FIGURE 1:**
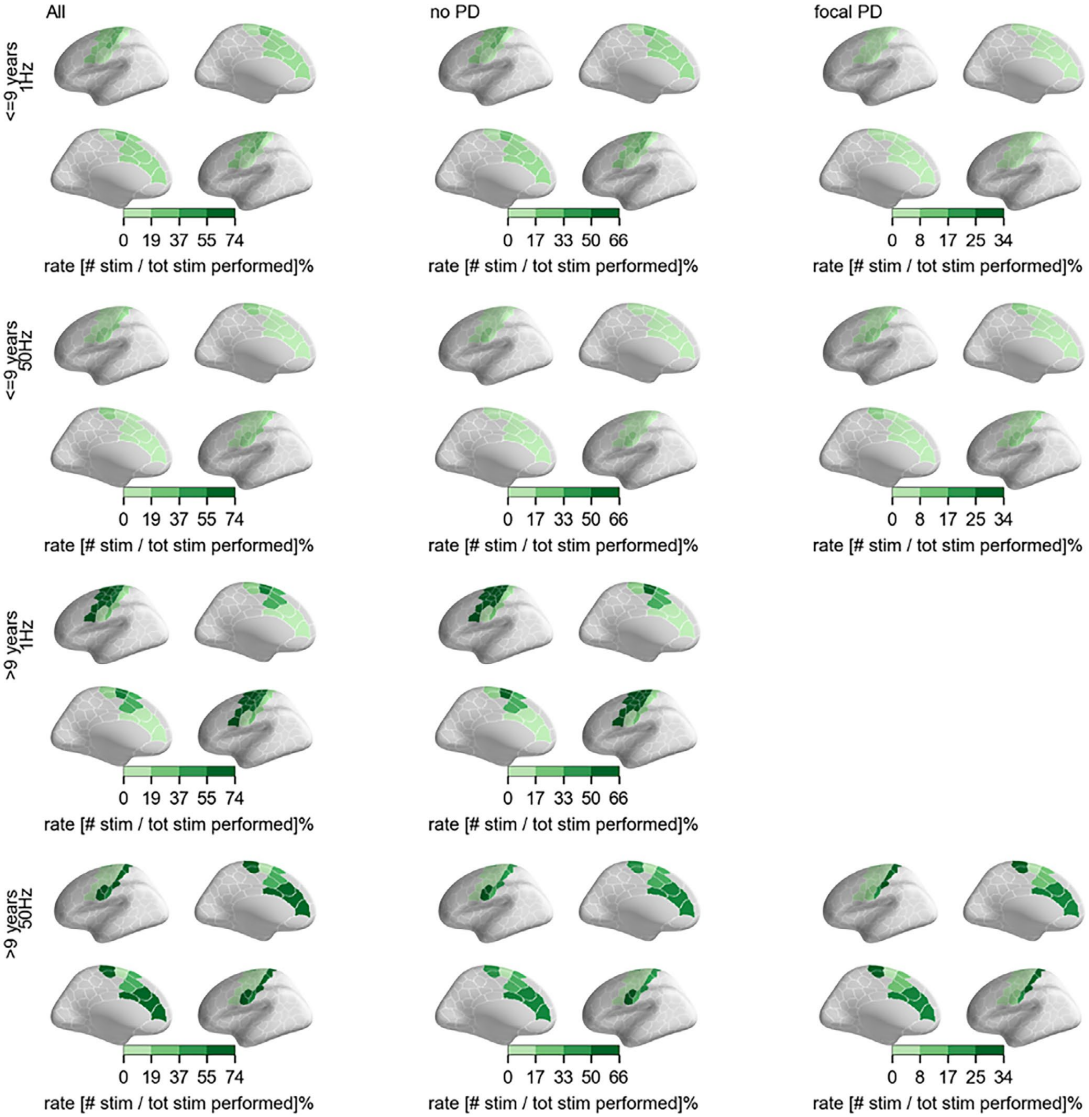
Responsiveness maps to intracerebral electrical stimulation (IES) by age and electrical effects elicited by IES. Lateral and medial surface projections show, for each sensory–motor areas of interest, the percentage of stimulations (stim) that evoked a clinical sign relative to the total stimulations (tot stim) delivered in that stratum (rate [# stim/tot stim performed] %; color bar). Column groups: all stimulations (IES with focal postdischarge [PD] plus IES without focal PD), no PD (IES without focal PD) and focal PD (IES with focal PD). Rows stratify by age (>9 years vs. ≤9 years). Regions: precentral gyrus, precentral operculum, postcentral operculum, postcentral gyrus, premotor cortex, supplementary sensory–motor area including the presupplementary part, anterior and central cingulate cortex. Gray indicates unsampled cortex.

In the subset without PD (*n* = 1641), the age effect remained significant (adjusted per‐year IRR = 1.13, 95% CI = 1.04–1.23, *p* = .005). When age was modeled categorically (>9 years vs. ≤9 years), the estimated effect was in the same direction but did not reach the prespecified significance threshold (*α* = .05, adjusted IRR = 1.90, 95% CI = .98–3.67, *p* = .057). IESs with focal PD were fewer than IES without PD. Within the PD‐positive subset, the clinical response rate was higher in >9‐year than ≤9‐year (median [IQR]: ≤9‐year group, 0 [0–1] across 50 IES vs. >9‐year group, 1 [0–2] across 59 IESs; Mann–Whitney test, *p* < .05; Figure 1). See Table 4 for full results for total IES and IES without PD. The results of the IES with focal PD are only presented in the text, as the small sample size did not allow for the use of the same statistical models used for the total IES cohort and IES without PD.

**TABLE 4:** Clinical response features of intracerebral electrical stimulation.

		IRR	OR	95% CI	*p* ^a^
Total IES	Clinical response rate increase per year	1.11	–	1.03–1.21	.007^a^
	Clinical response rate analyzed categorically	1.89	–	1.02–3.51	.044^a^
Total IES	Integrated response rate increase per year	–	1.03	.76–1.39	.869
	Integrated response rate analyzed categorically	–	3.69	.31–44.09	.303
Total IES	Multiple response rate increase per year	–	1.07	.91–1.26	.425
	Multiple response rate analyzed categorically	–	3.65	1.24–10.77	.019^a^
IES without PD	Clinical response rate increase per year	1.13	–	1.04–1.23	.005^a^
	Clinical response rate analyzed categorically	1.90	–	.98–3.67	.057
IES without PD	Integrated response rate increase per year	–	1.01	.73–1.39	.9661
	Integrated response rate analyzed categorically	–	4.07	.32–51.87	.279
IES without PD	Multiple response rate increase per year	–	1.17	.97–1.40	.097
	Multiple response rate analyzed categorically	–	5.30	1.38–20.36	.015^a^

*Note*: Age was modeled continuously (per 1‐year increase) and categorically as two age groups: >9 versus ≤9 years old.

Abbreviations: CI, confidence interval; IES, intracerebral electrical stimulation; IRR, incidence rate ratio; OR, odds ratio; PD, postdischarges.

^a^ A two‐sided *α* < .05 was considered statistically significant.

### 3.3 Integrated and multiple responses related to age

In the overall IES cohort (with and without PD), integrated responses did not vary with age, whether modeled continuously (per‐year odds ratio [OR] = 1.03, 95% CI = .76–1.39, *p* = .869) or categorically (>9‐year vs. ≤9‐year group OR = 3.69, 95% CI = .31–44.09, *p* = .303). By contrast, multiple responses were more likely in the >9‐year group when age was modeled categorically (adjusted OR = 3.65, 95% CI = 1.24–10.77, *p* = .019).

In the without‐PD subset (*n* = 1641), integrated responses again showed no association with age (adjusted per‐year OR = 1.01, 95% CI = .73–1.39, *p* = .961; adjusted categorical OR = 4.07, 95% CI = .32–51.87, *p* = .279). Multiple responses remained more likely in the >9‐year group (adjusted OR = 5.30, 95% CI = 1.38–20.36, *p* = .015).

In the PD‐positive subset, no associations with age were detected (integrated response: ≤9‐year 4 (8%) and >9‐year 4 [7%], *p* = 1.000; multiple response: ≤9‐year 3 (6%) and >9‐year 10 [17%], *p* = .073). See Table 4 for full results for total IES and IES without PD. The results for IES with focal PD are only presented in the text, as the small sample size did not allow for the use of the same statistical models used for the total IES cohort and IES without PD.

### 3.4 Analysis of the effect of area during IES

Using PREMOT as the reference, all other areas showed a significantly higher probability of eliciting a clinical response to IES. This gradient was present in both the overall cohort (IES with and without PD) and the without‐PD subset; the PD‐positive subset was not modeled due to small size. This analysis was performed adjusting for age, thus avoiding the influence of the lower response rate presented by children ≤9 years old.

Two response group emerged (adjusted IRR vs. PREMOT, *z*‐statistics in parentheses):

- Lower response group: ACC IRR = 1.687 (*p* = .016), PREC‐OP IRR = 2.618 (*p* < .001), POSTC‐OP IRR = 3.117 (*p* < .001).
- Higher response group: SSMA IRR = 7.569 (*p* < .001), PREC IRR = 9.725 (*p* < .001), POSTC IRR = 11.714 (*p* < .001).

Results were similar when restricting to IES without PD: ACC IRR = 1.81 (*p* = .020), PREC‐OP IRR = 2.30 (*p* = .002), POSTC‐OP IRR = 3.03 (*p* < .001), SSMA IRR = 8.45 (*p* < .001), PREC IRR = 11.08 (*p* < .001), POSTC IRR = 13.01 (*p* < .001; Figure 1).

## 4 DISCUSSION

This study investigated age‐related differences in responses to IES across primary and secondary sensory–motor cortical areas. Our results support an age effect on IES‐evoked clinical responsiveness, with older age associated with a higher likelihood of eliciting a clinical response. Age was analyzed both as a continuous predictor (per 1‐year increase) and using an exploratory, data‐driven stratification (>9 vs. ≤9 years); in both approaches, response rates increased with age, and multiple responses were more frequent in children >9 years old. Our working hypothesis originates from the clinical observations that younger patients exhibit reduced sensitivity to IES, as evidenced by a lower response rate during electrical stimulation. This is consistent with reports that seizure semiology is age‐dependent even when the EZ involves the same cortical structures,^22^, ^23^, ^24^, ^25^, ^26^ likely reflecting maturational differences in network integration and propagation. IES offers a unique window on cortical reactivity outside the epileptogenic network. In clinical SEEG, electrodes are often placed in eloquent cortex to delineate functional boundaries near the suspected EZ, and in regions hypothesized to belong to the network that later prove to be uninvolved. IES delivered to these nonepileptogenic contacts can approximate physiological conditions and thus reveal age‐dependent features of cortical functions. Because ASMs can blunt neuronal excitability^27^, ^28^ and lower cognitive level/neurodevelopmental delay has been linked to higher stimulation thresholds,^8^ age‐related differences in IES responsiveness could, in principle, be confounded. After adjusting for ASM load, cognitive level/neurodevelopmental delay, and stimulation frequency (1 vs. 50 Hz), the age‐related increase in clinical responsiveness remained significant, and the same pattern was observed when analyses were restricted to stimulations without focal PDs. Together, these findings indicate that the observed age effect is independent of medication burden, cognitive impairment, stimulation parameters, and epileptic activity.

Our finding that older children show a higher probability of IES‐elicited clinical responses is broadly compatible with developmental neuroimaging evidence indicating nonlinear maturation of functional connectome organization across childhood. In a large lifespan dataset, Sun et al. reported that the somatomotor system exhibits an early, coarse adultlike organization at the system level, whereas functional connectivity continues to change across childhood with ongoing refinement of network structure and system‐level properties. In this context, the inflection around ~9 years observed in our cohort should be interpreted as a data‐driven, clinically useful stratification—that is, a working hypothesis that warrants replication—rather than a definitive neurodevelopmental milestone. One plausible implication is that later stage network refinement may facilitate the propagation and integration of focal electrical perturbations, thereby increasing the likelihood that IES evokes observable clinical responses in older children.

Beyond connectome‐level organization, reduced responsiveness in younger children may also relate to white matter maturation. Less mature myelination could reduce the efficiency and synchrony of axonal conduction, even after neuronal activation, resulting in a greater attenuation of stimulation‐evoked activity. This interpretation is consistent with evidence that myelination of major white matter tracts, including long‐range association pathways, continues beyond childhood and through adolescence.^29^

A secondary aim was to examine age‐related differences in the complexity of clinical responses. We distinguished integrated motor responses, such as coordinated, purposeful behaviors (e.g., grasping, hand‐to‐mouth, postural adjustments), from multiple responses, that is, the co‐occurrence of signs across domains (e.g., motor plus language). We found that the likelihood of integrated motor patterns did not vary with age, whereas multiple responses were more frequent in older children. One interpretation is that integrated actions reflect stimulus‐evocable motor modules embedded in premotor/cingulate circuitry that are present early in life; in nonhuman primates, long‐train microstimulation of precentral/premotor cortex reliably elicits coordinated reach‐to‐grasp and hand‐to‐mouth actions,^30^ and in humans, SEEG stimulation of the anterior/midcingulate cortex elicits goal‐directed behaviors including reaching/grasping, consistent with an “actotopic” organization along the cingulate sulcus.^13^, ^31^

By contrast, multiple responses likely require coactivation across distributed cortical systems, which becomes more readily engaged as long‐range networks segregate and specialize through late childhood; large‐scale developmental connectome work shows continued refinement of somatomotor organization and rising system segregation across the first decade and into preadolescence, aligning with our higher probability of multiple responses in the >9 years group.^29^ This mechanistic dissociation is also compatible with the idea that integrated actions may tap innate/central patternlike motor solutions,^32^, ^33^ whereas multidomain responses reflect the later maturation of distributed cortical connectivity.

Ultimately, we investigated whether responsiveness varied among cortical regions after adjusting for age in the mixed‐effects models. Using PREMOT, the region with the lowest response rate, as a reference, we found a gradient of increasing responsiveness. Specifically, ACC, PREC‐OP, and POSTC‐OP exhibited lower responsiveness, whereas higher response rates were observed in POSTC, PREC, and SSMA. The identified hierarchy, characterized by reduced apparent responsiveness in the premotor, opercula, and cingulate areas, alongside heightened responsiveness in the primary sensory–motor cortex and SSMA, aligns with the recognized functional–anatomical structure of the human motor system. The primary sensory–motor areas possess extensive corticospinal and thalamocortical connections, making them more prone to produce time‐locked responses at lower stimulation thresholds.^8^ On the other hand, the premotor, opercula, and cingulate areas are more engaged in sensory–motor integration and action selection.^13^, ^34^ Thus, the effects of stimulation in these areas may be more complex and may require meticulous bedside examination for proper identification. These patterns are consistent with our a priori classification of the opercular cortices as secondary sensory–motor areas, despite their cytoarchitectonic proximity to the primary strip.^10^, ^12^, ^14^, ^15^, ^34^, ^35^, ^36^ Interestingly, the SSMA—although usually classified as a secondary motor area involved in movement planning—demonstrated response properties similar to those of primary areas, likely due to its pivotal role in motor initiation, execution, and maintaining movements.

## 5 LIMITATIONS AND CONCLUSIONS

Our study highlights how age significantly modulates cortical responsiveness to IES in pediatric populations, particularly within primary and secondary sensory–motor regions. The identification of a developmental cutoff around 9 years of age—corresponding to a marked increase in response rate and complexity—provides compelling evidence for ongoing sensorimotor network maturation beyond early childhood. This age‐related gradient was especially evident in the increased likelihood of multiple response patterns, suggesting progressive integration across functional domains with development.

Nonetheless, some limitations must be acknowledged. The age distribution within our cohort was not homogeneous, with a predominance of older children. This reflects the current clinical practice in which IC electrode implantation is more frequent in older pediatric patients. We analyzed only clinical effects and did not characterize electrophysiological responses. Future studies should combine corticocortical evoked potentials, stimulus‐locked markers (event‐related potentials/high‐gamma), and PD profiling with connectivity analyses to derive network‐level maps of IES propagation and excitability. Pairing these data with myelin‐sensitive imaging and a broader exploration of stimulation parameters (frequency, pulse width, train duration, current amplitude/charge per phase) would clarify whether age‐related differences in clinical responsiveness reflect parallel differences in electrophysiological excitability and large‐scale network recruitment. The variability in pulse width during 1‐Hz stimulation (1–3 ms), although limited (3 ms in ~6% of stimulations), could have influenced stimulation‐response relationships. In our dataset, electrode type/contact geometry and the bipolar adjacent‐contact configuration were uniform, and pulse‐width adjustments were clinically driven rather than systematically linked to age or stimulation site; however, we cannot exclude residual effects of parameter variability on response profiles.

Another point that was taken into account is patient collaboration. The study was designed based on the assumption that all patients were able to collaborate, although some of them were particularly young. However, some responses, particularly subjective ones, might have been underreported in younger children. An experienced examiner recognized these responses in several cases due to a sudden change in expression or behavior during the subsequent interview.

Moreover, we hope to expand our study with a multicenter study, as a larger sample would certainly provide greater statistical power.

Finally, our findings support the need for a shared framework in selecting stimulation parameters for functional mapping. Recognizing that certain cortical regions and age groups are intrinsically less likely to exhibit clinical responses can guide the adaptation of stimulation protocols, potentially improving the reliability of cortical mapping in children. These considerations are crucial for enhancing the safety and efficacy of SEEG‐guided interventions in the pediatric population.

## AUTHOR CONTRIBUTIONS

All authors contributed to the study conception and design. Material preparation, data collection, and analysis were performed by Giulia Nobile, Roberto Mai, Laura Tassi, Marta Ponzano, Lino Nobili, Piergiorgio d'Orio, Alessandro Consales, Luca Bosisio, Stefano Francione, Gabriele Arnulfo, and Maria Pia Sormani. The first draft of the manuscript was written by Giulia Nobile, Lino Nobili, Roberto Mai, Laura Tassi, and Marta Ponzano, and all authors commented on previous versions of the manuscript. All authors read and approved the final manuscript.

## CONFLICT OF INTEREST STATEMENT

L.N. has received support from Fidia Pharma, Idorsia, Jazz, and Eisai. The remaining authors have no conflicts of interest. We confirm that we have read the Journal's position on issues involved in ethical publication and affirm that this report is consistent with those guidelines.

## Supporting information


Figure S1.

Figure S2.

Table S1.

## Data Availability

Data may be provided to interested researchers upon reasonable request to the corresponding author.
