# Supplementary figures and images for: Pediatric sensorimotor cortical responsiveness to intracerebral stimulation during stereoelectroencephalographic monitoring: Age effects and area specificity
Source: Epilepsia. 2026 Apr 3;67(7):3590–601. doi: 10.1002/epi.70231 (PMC13360978; doi:10.1002/epi.70231)

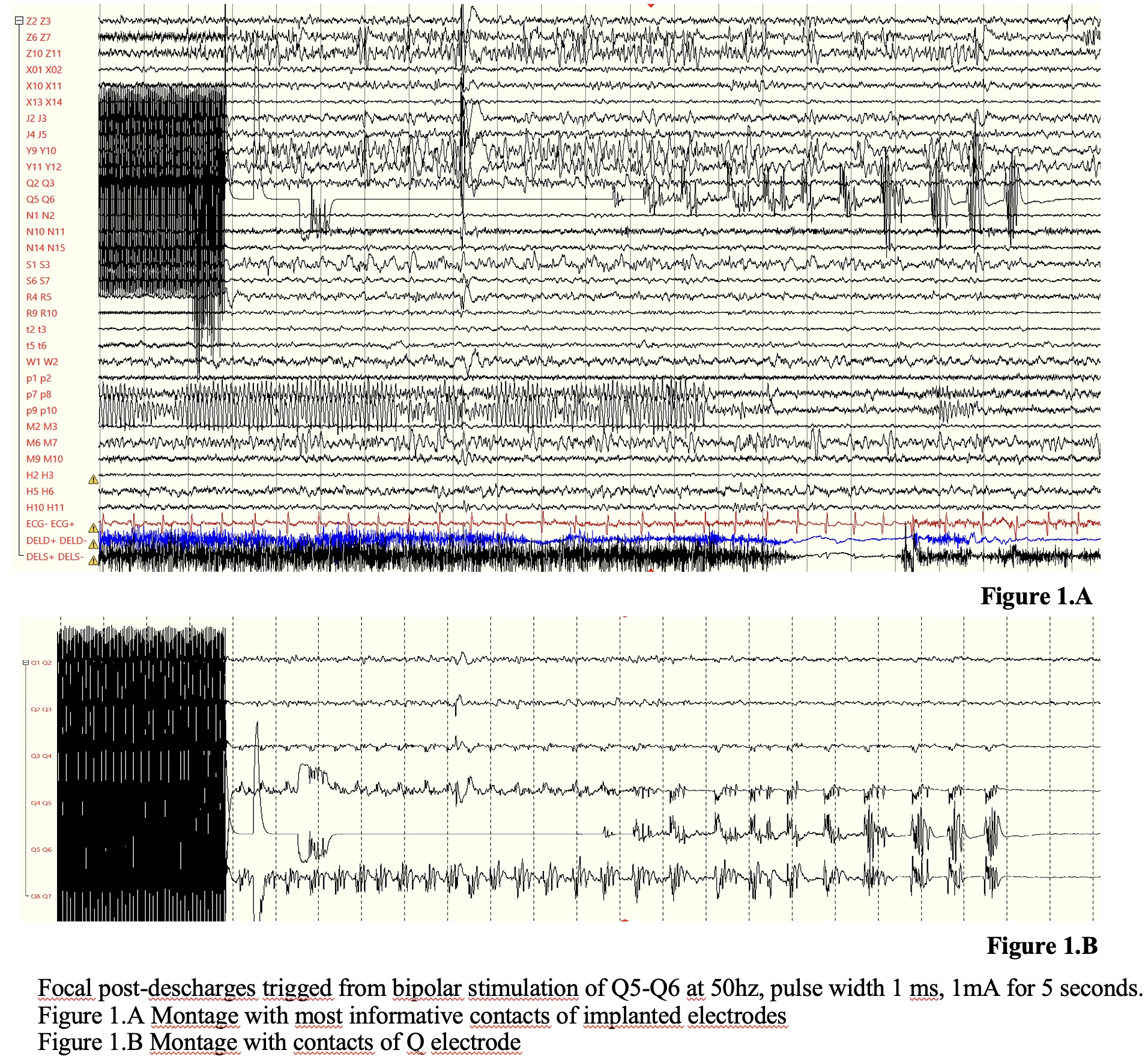

Supplement: Supplementary file 1 — Figure S1. [file EPI-67-3590-s002.jpg]

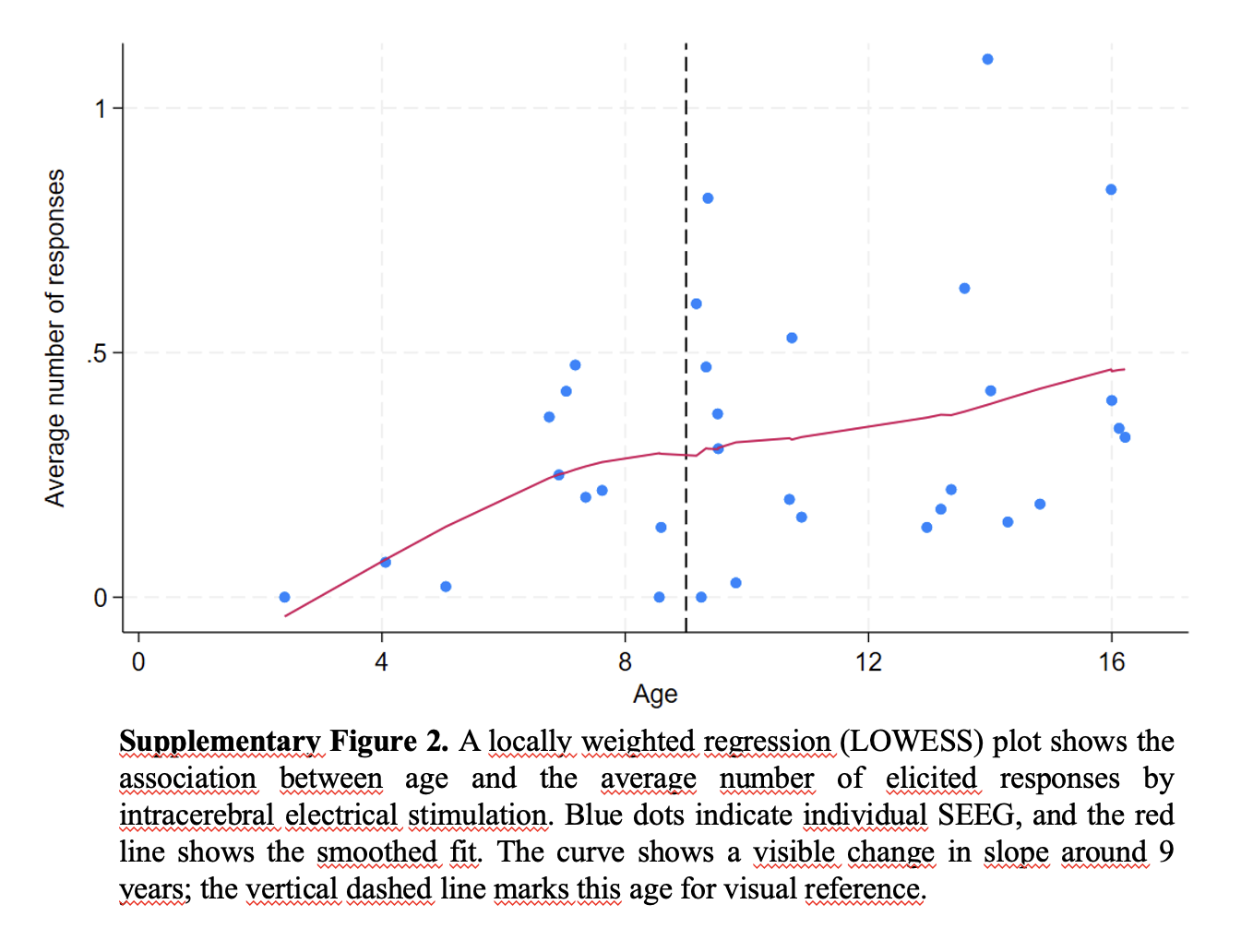

Supplement: Supplementary file 2 — Figure S2. [file EPI-67-3590-s003.tiff]
