# Supplementary material for: Pediatric sensorimotor cortical responsiveness to intracerebral stimulation during stereoelectroencephalographic monitoring: Age effects and area specificity
Source: Epilepsia. 2026 Apr 3;67(7):3590–601. doi: 10.1002/epi.70231 (PMC13360978; doi:10.1002/epi.70231)
Supplement: Supplementary file 3 — Table S1. [file EPI-67-3590-s001.docx]

**Supplementary Table 1. Derived charge metrics for intracerebral electrical stimulation (IES)**

| **Mode** | **Frequency** | **Current (mA)** | **Pulse width (ms)** | **Charge/phase (µC)** | **Charge density/phase (µC/cm²)** |
| --- | --- | --- | --- | --- | --- |
| Low-frequency IES | 1 Hz | 0.4–7 | 1 | 0.4–7 | 8.0–139 |
| Low-frequency IES | 1 Hz | 0.4–5 | 2 | 0.8–10 | 15.9–199 |
| Low-frequency IES | 1 Hz | 0.4–5 | 3 | 1.2–15 | 23.9–298 |
| High-frequency IES | 50 Hz | 0.2–5 | 1 | 0.2–5 | 4.0–99 |

Notes: Charge per phase (Q) was calculated as Q = I × PW, where I is current (mA) and PW is pulse width (ms); 1 mA × 1 ms = 1 µC. Charge density per phase (σ) was calculated as σ = Q / A using the geometric surface area of one cylindrical contact (excluding end caps): A = π × d × L, with electrode diameter d = 0.8 mm and contact length L = 2 mm (A = 0.05027 cm²). Inter-contact distance was 1.5 mm. Values are reported per phase per contact.
